# The challenge to disentangle age and recovery effects in research focusing on adolescent anorexia nervosa

**DOI:** 10.1038/s41398-022-02277-2

**Published:** 2022-12-13

**Authors:** Ilka Boehm, Holger Mohr, Stefan Ehrlich

**Affiliations:** 1 Division of Psychological and Social Medicine and Developmental Neurosciences, Translational Developmental Neuroscience Section, Technische Universität Dresden, University Hospital C.G. Carus, Dresden, Germany; 2 Department of Psychology, Technische Universität Dresden, Dresden, Germany; 3 Department of Child and Adolescent Psychiatry, Eating Disorder Treatment and Research Center, Technische Universität Dresden, Faculty of Medicine, University Hospital C. G. Carus, Dresden, Germany

**Keywords:** Psychiatric disorders, Neuroscience, Human behaviour

**Dear Editor,**

The authors of Boehm et al. [1] thank Levine [2] for the feedback to the recently published article in Translational psychiatry. Here, we would like to present our position to the two main points raised: 1) the appropriate statistical method to account for the age effect and 2) the potential of the representational similarity analysis (RSA) for the research question presented in Boehm et al. [1].

Anorexia nervosa (AN) is a mental disorder characterized by restriction of energy intake leading to low body weight mostly driven by intense fear of becoming fat and a distorted body image [3]. AN is the second most common mental disorder in adolescent girls and a serious condition with the highest mortality rate of any psychiatric disorder [4]. The highest incidence rates of AN are in the age range 14–19, with a peak in 14–15 years [5]. Beside, chronic undernutrition and years of illness-related deprivation may have their own consequences on brain structure and function [6]. Thus, it seems particularly important to investigate the disease in adolescence and to identify vulnerability markers to develop prevention and early intervention strategies. To distinguish markers that predispose towards the disorder from those that are simply a consequence of undernutrition, researchers typically recruit individuals who have recovered from AN (recAN) in addition to acutely ill underweight patients (acAN). This presents a challenge when studying adolescents. Participants who have gone through a recovery process are inherently older than adolescent patients experiencing their first episode. In other words, age is an unavoidable confounder when studying AN in adolescence and early adulthood.

There are several approaches with potential to address this challenge, which we discuss in the following. In this context we also address the criticism raised by Levine [2].

One common approach is to control for the influence of age by including a covariate. However, such use of an (M)ANCOVA is actually considered statistically inappropriate when the groups to be compared substantially differ as a function of the covariate. This point is made clearly in the widely referenced work of Miller and Chapman [7]. Another strategy, as presented in Boehm et al. [1], is the analysis of two different age-matched samples (acAN compared with age-matched healthy control (HC_acAN_); recAN compared with age-matched healthy control (HC_recAN_)).

Regarding this strategy, Levine [2] suggests focusing on the interaction contrast (acAN vs. HC_acAN_) vs. (recAN vs. HC_recAN_), where an effect driven by acAN vs. HC_acAN_ but not recAN vs. HC_recAN_ would support our interpretation (Fig. 1b in Levine (2022)). However, we found moderate evidence for the null-hypotheses of the contrast recAN vs. HC_recAN_ using Bayesian statistics, indicating that these groups are not meaningfully different from each other. This finding resolves concerns summarized in Levine (2022) in the Fig. 1a and Fig. 1b by providing evidence for the latter. We would like to add, that a very likely phenomenon to observe in similar studies, might be that both age as well as recovery come into play. In this case, the strategy suggested by Levine (2022) would not allow distinguishing both effects. Since we also have to assume that in adolescent AN maturational processes are delayed, estimating the age effect based on younger and older HC [7] maybe also considered insufficient. Ultimately, only time-consuming longitudinal studies remain to isolate the recovery effect and identify predisposing factors.

With respect to the second point raised by Levine [2], we share the enthusiasm for RSA as a valuable approach to characterize multiple neural representations by distance measures of response patterns to describe a neural process. The procedure Levine [2] has proposed to support our tentative interpretation in Boehm et al. [1] sounds very interesting indeed. However, we would like to emphasize that the study design as presented in Boehm et al. [1] is not well-suited for this approach. To conduct a RSA study in order to provide a fine-grained characterization of the “food representational space” one would have to cover as many food categories as possible. Moreover, the experimental design should ideally be an event-related with sufficiently long inter-trial intervals, combined with single-trial analyses, see for example Visser et al. [8].

In closing, we thank Dr. Levine for opening the discussion about the challenges to address age effects when investigating adolescent psychiatric conditions and for pointing out potential statistical approaches which may help following up on our findings.
